# Glycinebetaine Improves Photosynthetic Performance and Antioxidant Defense in Barley Under Water Deficit Conditions

**DOI:** 10.3390/biom16030372

**Published:** 2026-03-02

**Authors:** Kh. Armane Alam, Shanjida Karim, Sharmin Sultana, Ashim Kumar Das, Apple Mahmud, Md. Abiar Rahman, Md. Motaher Hossain, Yeasin Arafat, Shohana Parvin, Moon-Sub Lee

**Affiliations:** 1 Department of Agroforestry and Environment, Gazipur Agricultural University, Gazipur 1706, Bangladesh; khondokararman516@gmail.com (K.A.A.); shanjida6123@stu.gau.edu.bd (S.K.); abiar@gau.edu.bd (M.A.R.); 2 Department of Crop, Soil and Environmental Sciences, Auburn University, Auburn, AL 36849, USA; szs0438@auburn.edu; 3 Department of Applied Biosciences, College of Agriculture and Life Sciences, Kyungpook National University, Daegu 41566, Republic of Korea; ashim@knu.ac.kr; 4 Department of Agronomy, Gazipur Agricultural University, Gazipur 1706, Bangladesh; apple885@gau.edu.bd; 5 Department of Plant Pathology, Gazipur Agricultural University, Gazipur 1706, Bangladesh; hossainmm@gau.edu.bd; 6 Faculty of Agriculture, Gazipur Agricultural University, Gazipur 1706, Bangladesh; ta9476477@gmail.com; 7 Department of Crop Science, Chungbuk National University, Cheongju 28644, Republic of Korea

**Keywords:** antioxidant activity, barley, drought stress, foliar application, glycinebetaine

## Abstract

Drought stress poses a serious threat to global agriculture, affecting plant growth, physiology, and biochemical processes, thereby impacting food security. Supplementation of phytohormones regulates plant physiological processes and improves tolerance to abiotic stress. In our study, we applied glycine betaine (GB), a non-toxic, highly soluble signaling molecule that plays an important role in protecting plants from environmental stress. To assess the role of with and without exogenous GB against fourteen days of prolonged drought stress (60% and 30% field capacity) on two high-yielding barley varieties, *BARI barley-6* (sensitive) and *BARI barley-9* (tolerant), with control plants were maintained at 90% field capacity. Results showed that both varieties exhibited a significant reduction in biomass, leaf relative water content, and photosynthetic activity under drought stress, while increasing the accumulation of proline and ROS, which indicates oxidative damage. In contrast, foliar application of GB improved growth, photosynthetic pigments, and net photosynthetic rate. It also helped to detoxify ROS by boosting the activities of antioxidant enzymes such as CAT, APX, POD, and GST while upregulating secondary metabolites like phenolic and flavonoid contents, maintaining membrane integrity, and regulating osmotic balance under water-deficient conditions. Overall, GB enhanced the drought tolerance of both barley varieties by modulating various physiological and biochemical processes. Our findings provide insights into GB-induced adaptation mechanisms in plants that combat water scarcity and may help to develop drought-resilient crops.

## 1. Introduction

Drought is widely recognized as the most devastating abiotic stress affecting global agriculture, resulting in significant yield reductions across cereal crops. Its increasing frequency and severity under climate change threaten both food security and ecosystem balance [1,2]. Over the last two decades, natural disasters accounted for 23% of losses in the agricultural sector, with drought alone causing over 65% of those losses [3]. Prolonged water deficit leads to stomatal closure, disruption of photosynthetic efficiency, inhibition of leaf expansion, decline in plant height, biomass, and grain yield across major cereals such as maize, wheat, and barley [4,5,6]. In response to these conditions, plant cells trigger an excessive generation of reactive oxygen species (ROS), such as hydrogen peroxide (H_2_O_2_) and superoxide radicals, causing lipid peroxidation, membrane leakage, and nucleic acid damage [7]. To counter the cellular damage, plants activate a complex antioxidant defense network comprising enzymatic scavengers–superoxide dismutase (SOD), catalase (CAT), and peroxidases (POD), as well as non-enzymatic metabolites [8,9]. In addition, plants alter diverse signaling molecules, osmolytes, and hormonal levels in their tissues under stress conditions, thereby enhancing their tolerance efficiency. Recently, exogenous application of these compounds has also been used as an alternative approach to contribute to stress tolerance.

Foliar application is one of the approaches that has emerged as a rapid, efficient, and environmentally safe method for delivering stress-alleviating compounds directly to leaves, where rapid uptake enhances plant metabolism under adverse conditions [10,11]. It enables immediate physiological adjustments such as osmotic balance restoration and antioxidant activation. Unlike soil supplementation, foliar sprays bypass rhizosphere constraints such as reduced root uptake under drought, allowing rapid absorption through cuticular and stomatal pathways for efficient translocation and stress mitigation [12].

Glycinebetaine (GB; *N,N,N*-trimethylglycine) is a highly soluble, non-toxic, zwitterionic quaternary ammonium compound that accumulates in plants when exposed to stresses [13,14,15,16,17]. GB is synthesized through the choline oxidation pathway involving choline monooxygenase (CMO) and betaine aldehyde dehydrogenase (BADH), which convert choline to betaine aldehyde and then to GB [14,15,18]. These chloroplast-localized enzymes depend on ferredoxin and NAD^+^ redox systems, linking the GB synthesis pathway to photosynthetic electron transport [19]. Its accumulation is actively regulated by stress-related signal transduction pathways; for example, jasmonate (JA)-mediated signaling upregulates CMO and BADH expression in watermelon under osmotic stress, demonstrating the integration of hormonal and metabolic regulation [20,21]. The GB application contributes to stress tolerance by maintaining cellular osmotic balance through the reduction in cytosolic water potential, stabilizing proteins and membrane lipids, particularly within photosystem II complexes, and protecting the photosynthetic apparatus from oxidative and photo-inhibitory damage [14]. Furthermore, exogenous GB supplementation through foliar sprays or seed priming similarly enhances stress tolerance in species with low endogenous GB levels by improving ion homeostasis, upregulating H^+^-ATPase and Na^+^/H^+^ antiporter genes, and modulating abscisic acid (ABA) and JA-dependent signaling pathways [22,23]. Collectively, endogenous synthesis or exogenous supplementation of GB strengthens osmoprotection, redox balance, and overall plant tolerance to multiple abiotic stresses.

Barley (*Hordeum vulgare* L.) is the oldest crop and ranks 4th among cereal crops in the world. Although barley is inherently endurant to harsh environments, severe drought has decreased barley production by more than 50% [24]. Previous studies showing positive effects of GB on maize, rice, pepper, and tomato under various stresses, providing us a clue to expand the application of GB-mediated stress tolerance in barley. Additionally, previous studies have poorly examined how GB application affects plants under drought stress. Therefore, we not only determine the effect of GB on growth and development but also provide a defense mechanism that explains how foliar GB supports the plant’s photosynthetic and detoxification systems when water is scarce. Specifically, we elucidate the effect of GB under two water-deficit conditions on barley growth, photosynthetic performance, ROS detoxification, and osmotic regulation. Addressing this gap could improve our understanding of GB’s role in cereal drought adaptation and support the development of agronomic strategies for barley in water-limited environments.

## 2. Materials and Methods

### 2.1. Plant Materials and Experimental Design

Two high-yielding barley varieties, namely *BARI Barley-6* (2.5–2.75 ton ha^−1^) (susceptible) and *BARI Barley-9* (2.2–2.4 ton ha^−1^) (tolerant) (https://dhcrop.bsmrau.net/bari-barley/, accessed on 12 November 2025), were used to assess the efficacy of GB in mitigating drought-induced adverse effects. Healthy seeds were collected from the Plant Genetic Resource Center (PGRC) at the Bangladesh Agricultural Research Institute (BARI), Gazipur, Bangladesh. The experiment was conducted under a polyhouse from January to February 2025 to reduce the risk of rain. During the experimental period, temperatures ranged from 13 °C to 26 °C. Briefly, *BARI Barley-6* and *BARI Barley-9* were sterilized using sodium hypochlorite (5%, *v*/*v*) for 20 min and subsequently rinsed five times with distilled H_2_O (dH_2_O). The seeds were pre-soaked in dH_2_O for eight hours at room temperature in the dark to accelerate germination. Then, twenty-five seeds of each variety were sown in plastic pots (height × diameter = 12.5 cm × 13 cm), each containing 750 g of soil composed of a 2:1 weight ratio of cow manure. The soil pH was about 6.4, and it was treated with pesticide Furadan (3.0 g pot^−1^) to prevent soil-borne diseases. After germination, we meticulously selected and kept the twelve most uniform seedlings in each pot.

Fourteen days after germination, plants were subjected to drought exposure; all pots were divided into three groups: control (CK) (~90% field capacity), moderate drought (~60% field capacity), and severe drought (~30% field capacity) treatments following the gravimetric method. Briefly, the pots were thoroughly watered first, allowing excess water to drain from the bottom and leaving the soil saturated. Each pot was weighed daily, and this weight was used to determine the amount of water needed to achieve a specific drought-stress level based on the percentage of field capacity. Water was then added as needed to maintain the target soil moisture throughout the treatment. The gravimetric method allowed precise control of soil water content to impose drought stress for 2 weeks [25]. The approximate soil moisture content at different FC was determined by the difference between the soil weight after drainage and the soil weight after oven drying at 100 °C for 24 h. Each group was subdivided into two, with or without GB treatment. In brief, the whole experimental setup consisted of six treatment groups: (i) without GB-supplemented control plants (~90% FC; CK), (ii) 100 mM GB-supplemented control plants (~90% FC; CK + GB), (iii) without GB-supplemented drought stress plants (~60% FC; D1), (iv) 100 mM GB-supplemented drought-stressed plants (~60% FC; D1 + GB), (v) without GB-supplemented drought stress plants (~30% FC; D2), and (vi) 100 mM GB-supplemented drought-stressed plants (~30% FC; D2 + GB). The dose and preparation of exogenous 100 mM GB (Sigma Aldrich CAS No: 107-43-7, St. Louis, MO, USA) were selected based on a previous report by Shemi et al. [26]. Tween-20 (0.2%; *v*/*v*) was added as a surfactant to the GB solution and water to ensure maximum adherence of the treatment solutions to the leaf surface. Plants were sprayed with 50 mL of water or GB per pot between 9:30 and 10:00 a.m. All morphological, physiological, and biochemical analyses were performed on leaves freshly harvested from 28-day-old plants. This study used a randomized complete block design with five replications per treatment.

### 2.2. Growth Attributes and Leaf Features

After two weeks, we evaluated the effects of drought stress on barley phenotypes by photographing the plants using a Canon EOS 90D digital camera (Canon Inc. Operations, Tokyo, Japan) following the drought stress treatment. We then randomly selected three plants from each treatment group to assess growth traits, including plant height, root length, and the fresh and dry weights of both shoots and roots. To measure plant height, we used a ruler to determine the distance from the base to the apex of the primary stem. The roots of both the well-watered and drought-treated plants were carefully removed from the soil and thoroughly cleaned with tap water to eliminate any soil particles. The shoot and root samples were placed in an oven at 80 °C for 72 h to measure their dry weights (DWs).

### 2.3. Quantifying Chlorophylls and Carotenoids

Chlorophyll a (Chl *a*), chlorophyll b (Chl *b*), total chlorophylls (Chls), and carotenoids were quantified from supernatants extracted with 80% (*v*/*v*) acetone (Fisher Scientific, Loughborough, UK, CAS No: 35-16-7) using a GENESYS 10S spectrophotometer (Thermo Scientific, San Jose, CA, USA). The levels of Chl *a*, Chl *b*, Chls, and carotenoids were calculated according to the formulas established by Arnon [27] and Lichtenthaler and Wellburn [28].

### 2.4. Determination of Photosynthetic Gas Exchange Attributes

The net photosynthetic rate (*Pn*), leaf temperature (*LT*), transpiration rate (*E*), and stomatal conductance (*gs*) of the barley plants were measured using a portable LI-6400XT instrument (LI-COR Inc., Lincoln, NE, USA) under ambient sunlight conditions between 11:00 a.m. and 1:00 p.m.

### 2.5. Assessing Reactive Oxygen Species in Leaves, Quantifying Hydrogen Peroxide, Malondialdehyde, and Electrolyte Leakage Levels

Solutions of nitroblue tetrazolium (NBT) and 3,3′-diaminobenzidine (DAB) were used to visualize the accumulation of superoxide (O_2_^•−^) and hydrogen peroxide (H_2_O_2_) in newly collected third leaves of barley seedlings, as outlined by Das et al. [29]. Additionally, the GENESYS 10S spectrophotometer (Thermo Scientific, San Jose, CA, USA) was used to measure the concentrations of H_2_O_2_ and malondialdehyde (MDA) in the third leaf, following the procedures outlined by Yu et al. [30] and Kim et al. [31], respectively. Electrolyte leakage (EL) in barley leaves was assessed using an electrical conductivity meter (HORIBA Twin Model B-173, Kyoto, Japan), following Kim et al. [31]. We immersed 0.2 g of leaf tissue in 20 mL of tap water to measure initial electrical conductivity (EC1). After heating at 100 °C for 30 min, cooling, and measuring again (EC2), electrolyte leakage (EL) was calculated as EL (%) = (EC1/EC2) × 100.

### 2.6. Determination of Enzymatic and Non-Enzymatic Antioxidant Activities

Enzyme extracts were prepared from the third leaves of barley seedlings, and the activities of antioxidant enzymes, specifically catalase (CAT) (EC: 1.11.1.6), glutathione S-transferases (GST) (EC: 2.5.1.18), ascorbate peroxidase (APX) (EC: 1.11.1.11), and peroxidase (POD) (EC: 1.11.1.7), were assessed as outlined by Rahman et al. [32]. The total phenolic and flavonoid contents in fresh samples of the third leaf were quantified according to the methodology described by Das et al. [29] to assess the non-enzymatic antioxidant levels.

### 2.7. Determination of Leaf Relative Water Content, Proline, Total Soluble Sugar, Total Free Amino Acid, and Total Carbohydrates

Relative water content was measured following the technique established by Das et al. [29]. The proline (Pro) level was assessed using the acid ninhydrin method as described by Bates et al. [33]. The methodologies outlined by Somogyi [34] and Lee and Takahashi [35] were employed to quantify the levels of total soluble sugar (TSS) and total free amino acids (TFAA), respectively. The methodology of Dubois et al. [36] was used to evaluate the concentration of total carbohydrate (Carb).

### 2.8. Statistical Analysis

The acquired data were evaluated using a two-way analysis of variance (ANOVA) in RStudio (version: 2025.09.2+418). Statistically significant differences (*p* < 0.05) across diverse treatments were indicated by distinct letters, utilizing RStudio software. Three biological replicates (*n* = 3) were used to calculate the values (means ± SEs) for each treatment, which are illustrated in Figures and Tables.

## 3. Results

### 3.1. GB Enhances the Growth and Morphological Attributes of Barley Plants Under Water Deficit Conditions

**Figure 1:**
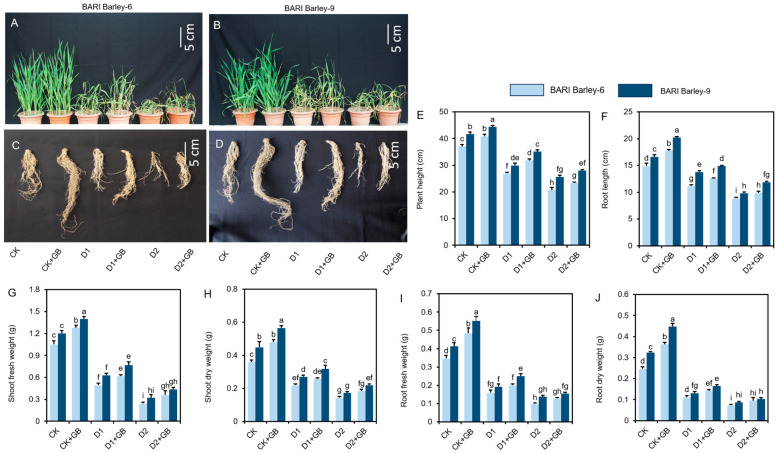
Effects of glycine betaine (GB) on the biomass of (**A**,**C**) *BARI Barley-6* and (**B**,**D**) *BARI Barley-9* plants subjected to drought stress (DS) for a period of two weeks. (**E**) Plant height, (**F**) root length, (**G**) shoot fresh weight, (**H**) shoot dry weight, (**I**) root fresh weight, and (**J**) root dry weight of barley plants exposed to drought for two weeks with and without GB treatments. Statistically significant differences (*p* < 0.05) among treatments are indicated by different letters. CK, without GB-supplemented control plants; CK + GB, 100 mM GB-supplemented control plants; D1, without GB-supplemented drought stress plants; D1 + GB, 100 mM GB-supplemented drought-stressed plants; D2, without GB-supplemented drought stress plants; and D2 + GB, 100 mM GB-supplemented drought-stressed plants.

To explore the effect of GB in alleviating the drought-induced adverse impacts on growth parameters, we documented plant phenotypes, shoot height, shoot fresh weight, shoot dry weight, root length, root fresh weight, and root dry weight. When barley varieties were exposed to drought stress for two weeks, their phenotypic appearance was significantly changed. In comparison with corresponding ‘CK’ plants, drought plants showed limited development, thinner leaves, and a mild browning of the leaves (Figure 1A–D). Intriguingly, ‘D1 + GB’- and ‘D2 + GB’-treated barley plants drastically diminished the drought-induced toxicity and significantly improved the phenotypic appearance of both *BARI Barley-6* and *-9* varieties, when compared with the corresponding ‘D1’ and ‘D2’ plants (Figure 1A–D). Nevertheless, in relation to ‘CK’ plants, ‘GB-treated’ plants improved the visual appearance of both *BARI barley-6* and *-9* varieties under non-stressed conditions (Figure 1A–D).

Compared with ‘CK’ plants, drought-stressed *BARI Barley-6* and *-9* plants displayed substantial reductions in plant height, root length, shoot fresh and dry weight, and root fresh and dry weight (Figure 1E–J). However, compared with ‘D1’ and ‘D2’ plants, ‘D1 + GB’ and ‘D2 + GB’ plants exhibited improvement in these morphological traits (Figure 1E–J). A parallel enhancement was noticed in ‘GB-treated’ *BARI Barley-6* and *-9* ‘GB’ plants, when compared with that of corresponding ‘CK’ plants (Figure 1E–J). Moreover, the tolerant variety *BARI Barley-9* outperformed in endurance under drought conditions over susceptible variety *BARI Barley-6* with GB supplementation (Figure 1A–J). Collectively, our results suggest that the ‘GB’ application is a promising approach to improving barley fitness under water deficit stress.

### 3.2. GB Safeguarded Photosynthetic Pigments and Gas Exchange Features in Response to Water Deficit Conditions

**Figure 2:**
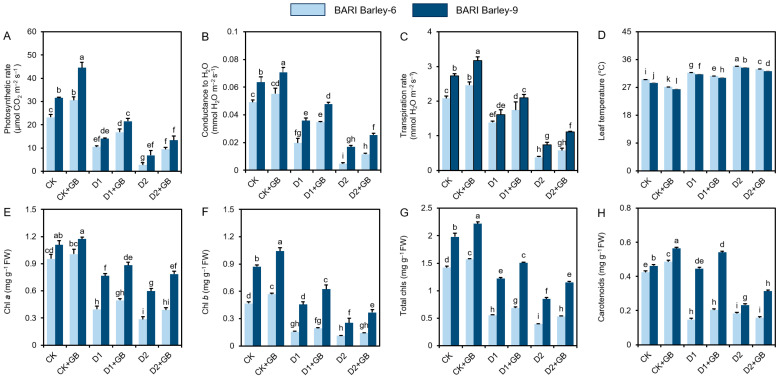
Effects of exogenously applied 100 mM GB on the levels of (**A**) net photosynthetic rate, (**B**) stomatal conductance to water, (**C**) transpiration rate, (**D**) intercellular CO_2_ concentration, (**E**) Chl *a*, (**F**) Chl *b*, (**G**) total Chls, and (**H**) carotenoids in the leaves of barley varieties exposed to two weeks of drought stress. Statistically significant differences (*p* < 0.05) among treatments are indicated by different letters. Chl, Chlorophyll; FW, Fresh weight; CK, without GB-supplemented control plants; CK + GB, 100 mM GB-supplemented control plants; D1, without GB-supplemented drought stress plants; D1 + GB, 100 mM GB-supplemented drought-stressed plants; D2, without GB-supplemented drought stress plants; and D2 + GB, 100 mM GB-supplemented drought-stressed plants.

To better understand the impacts of GB on the protection of photosynthetic pigments and gas exchange features under drought stress, we assessed Chl *a*, Chl *b*, total Chls, carotenoids, photosynthetic activity, stomatal conductance to water, transpiration rate, and leaf temperature levels in barley leaves. Compared with ‘CK’ plants, ‘D1’ and ‘D2’ *BARI Barley-6* and *-9* plants displayed a significant decrease in the levels of photosynthetic rate (*Pn*), stomatal conductance (*gs*), and transpiration rate (*E*), along with increases in leaf temperature (*LT*) (Figure 2A–D). In contrast, supplementation of ‘GB’ showed substantial improvements in *Pn*, *gs*, and *E*, while *LT* decreased in both varieties (Figure 2A–D).

Relative to the ‘CK’ plants, ‘D1’ and ‘D2’ plants exhibited significant decreases in the levels of Chl *a*, Chl *b*, total Chls, and carotenoids in both barley varieties (Figure 2E–H). By comparison, ‘GB-treated’ plants exhibited high levels of Chl *a*, Chl *b*, total Chls, and Caro in the leaves of ‘D1 + GB’ and ‘D2 + GB’ *BARI Barley-6* and *-9* plants, when contrasted with the corresponding ‘Drought’ plants (Figure 2E–H). GB-treated *BARI Barley-6* and *-9* plants showed significant increases in Chl *a*, Chl *b*, total Chls, and carotenoids, compared with CK plants (Figure 2A–H). Our findings indicated that supplementation of ‘GB’ boosted the photosynthetic pigment content in both varieties, with *BARI Barley-9* exhibiting higher protection of photosynthetic pigments than *BARI Barley-6* under drought conditions.

### 3.3. GB Reduced Oxidative Damage in Barley Leaves Under Water Deficit Conditions

**Figure 3:**
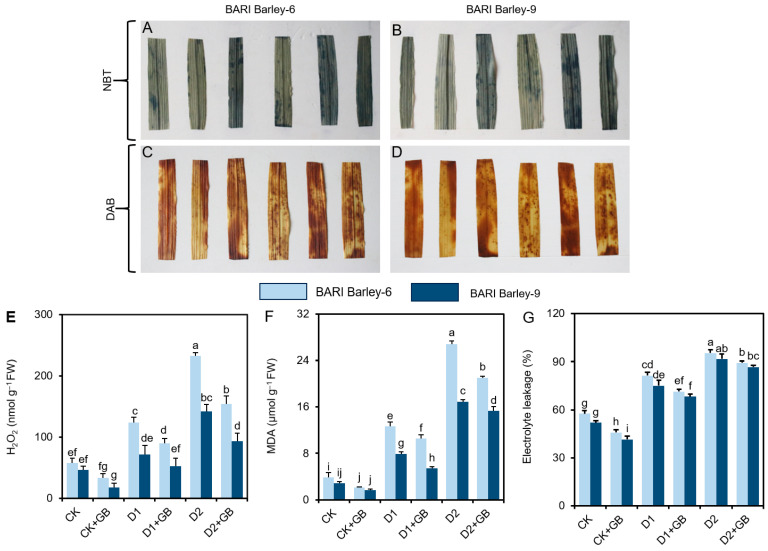
Effects of exogenously applied 100 mM GB on (**A**,**B**) nitroblue tetrazolium (NBT) staining for the detection of superoxide (O_2_^•−^), (**C**,**D**) diaminobenzidine (DAB)-staining for the detection of H_2_O_2_, (**E**) H_2_O_2_ content, (**F**) MDA content, and (**G**) electrolyte leakage in the leaves of barley seedlings exposed to two weeks of drought stress. Statistically significant differences (*p* < 0.05) among treatments are indicated by different letters. H_2_O_2_, Hydrogen peroxide; MDA, Malondialdehyde; FW, Fresh weight; CK, without GB-supplemented control plants; CK + GB, 100 mM GB-supplemented control plants; D1, without GB-supplemented drought stress plants; D1 + GB, 100 mM GB-supplemented drought-stressed plants; D2, without GB-supplemented drought stress plants; and D2 + GB, 100 mM GB-supplemented drought-stressed plants.

Relative to the ‘CK’ plant leaves, staining of the leaves of ‘D1’ and ‘D2’ plants with nitro blue tetrazolium (NBT) for superoxide (O_2_^•−^) and 3,3′-diaminobenzidine (DAB) for hydrogen peroxide (H_2_O_2_) resulted in the development of deeper blue and dark brown spots, respectively (Figure 3A,B). In comparison, ‘D1 + GB’ and ‘D2 + GB’ plant leaves showed a notable reduction in the accumulation of O_2_^•−^ and H_2_O_2_, when contrasted with the corresponding ‘D1’ and ‘D2’ plant leaves (Figure 3A–D).

More specifically, we measured the concentrations of H_2_O_2_ and MDA, as well as the levels of EL in barley leaves. ‘D1’ and ‘D2’ plants of *BARI Barley-6* and *-9* displayed notable augmentation in the levels of H_2_O_2_, MDA, and EL when compared with respective ‘CK’ plants (Figure 3E–G). Conversely, supplementation of ‘GB’ to *BARI Barley-6* and *-9* plants substantially attenuated these H_2_O_2_, MDA, and EL levels in ‘D1 + GB’ and ‘D2 + GB’ plants, relative to their corresponding ‘D1’ and ‘D2’ treatments (Figure 3E–G). Additionally, GB-treated control plants (CK + GB) also showed a substantial reduction in these levels compared to the untreated CK plants (Figure 3E–G). Furthermore, treated plants were more effective in minimizing the burden of ROS-mediated oxidative damage in the tolerant *BARI Barley-9* variety compared to the sensitive *BARI Barley-6* variety under both ‘D1’ and ‘D2’ stress.

### 3.4. GB Enhanced Antioxidant Defense Responses Under Water-Deficient Conditions

**Figure 4:**
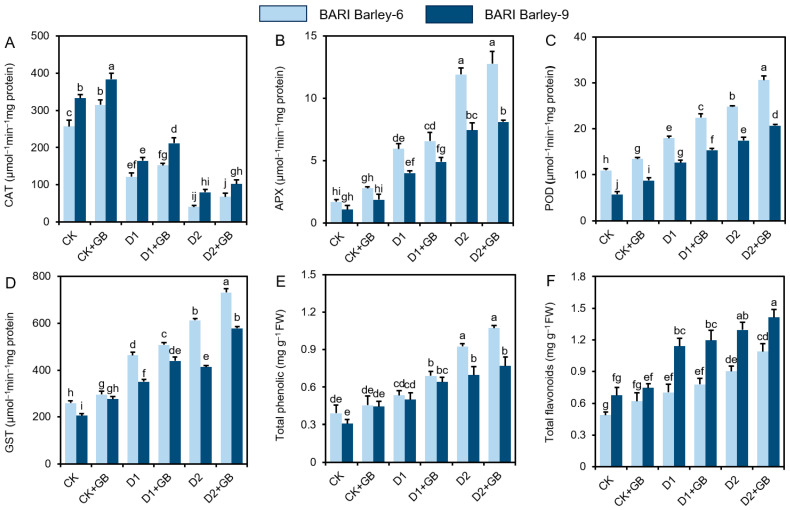
Effects of exogenously applied 100 mM GB on the activities of (**A**) catalase (CAT), (**B**) ascorbate peroxidase (APX), (**C**) peroxidase (POD), (**D**) glutathione S-transferase (GST), and the levels of (**E**) total phenolic, (**F**) total flavonoids in the leaves of barley seedlings exposed to two weeks of drought stress. Statistically significant differences (*p* < 0.05) among treatments are indicated by different letters. FW, Fresh weight; CK, without GB-supplemented control plants; CK + GB, 100 mM GB-supplemented control plants; D1, without GB-supplemented drought stress plants; D1 + GB, 100 mM GB-supplemented drought-stressed plants; D2, without GB-supplemented drought stress plants; and D2 + GB, 100 mM GB-supplemented drought-stressed plants.

To assess how GB modulates the regulation of antioxidant defense systems, we evaluated total flavonoids and phenolic compounds, as well as the activities of key antioxidant enzymes, including CAT, POD, APX, and GST (Figure 4A–D). Despite lower CAT activities under ‘D1’ and ‘D2’ conditions, *BARI Barley-6* and *-9* plants exhibited higher GST, POD, and APX activities (Figure 4A–D). GB supplementation in ‘D1 + GB’ and ‘D2 + GB’ plants significantly further enhanced enzyme activities compared with the corresponding ‘D1’ and ‘D2’ plants, while CAT and APX showed an insignificant difference (Figure 4A–D). Moreover, GB-treated both barley variety plants significantly showed higher CAT, POD, APX, and GST activities except APX than ‘CK’ plants (Figure 4A–D).

‘D1’ and ‘D2’ stress increased total flavonoid and phenolic component concentrations in *BARI Barley-6* and *-9* plants, respectively, compared with ‘CK’ plants (Figure 4E,F). Supplementation of ‘GB’ further increased both total flavonoids and phenolic levels in ‘D1 + GB’ and ‘D2 + GB’ plants for both *BARI Barley-6* and *-9*, compared with the corresponding drought-stressed plants, although they were non-significant (Figure 4E,F). Furthermore, GB-treated barley showed higher concentrations of total flavonoid and phenolic components than ‘CK’ plants without a significant difference (Figure 4E,F). Results of our study demonstrated that supplementation of ‘GB’ effectively boosted both antioxidant and non-antioxidant activities in both varieties, where *BARI Barley-9* exhibited higher antioxidant activity than *BARI Barley-6* under drought conditions (Figure 4A–F).

### 3.5. GB Improved the Levels of Osmoprotectants in Barley Leaves Under Water Deficit Conditions

**Figure 5:**
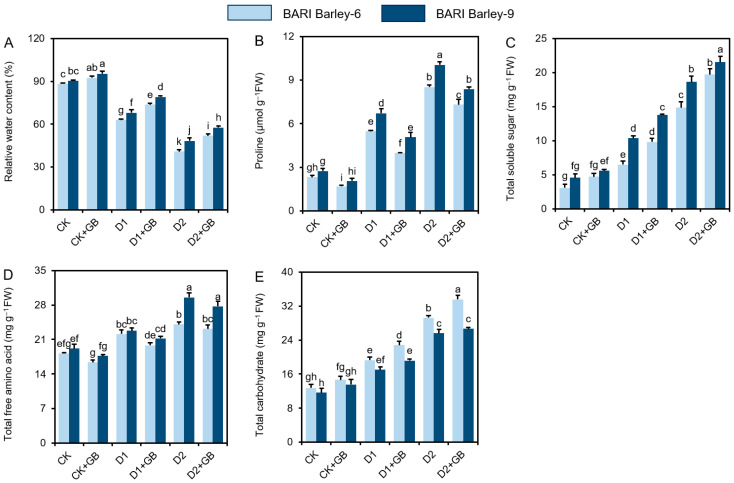
Effects of exogenously applied 100 mM GB on the levels of (**A**) relative water content, (**B**) proline, (**C**) total soluble sugar, (**D**) total free amino acid, and (**E**) total carbohydrate in the leaves of barley plants exposed to two weeks of drought stress. The statistically significant differences (*p* < 0.05) among various treatments are indicated by different letters. FW, Fresh weight; CK, without GB-supplemented control plants; CK + GB, 100 mM GB-supplemented control plants; D1, without GB-supplemented drought-stressed plants; D1 + GB, 100 mM GB-supplemented drought-stressed plants; D2, without GB-supplemented drought-stressed plants; and D2 + GB, 100 mM GB-supplemented drought-stressed plants.

*BARI Barley-6* and *-9* ‘D1’ and ‘D2’ plants exhibited drastically lower values of leaf RWC compared to ‘CK’ plants (Figure 5A). In contrast, the ‘GB’ application raised the levels of leaf RWC in *BARI Barley-6* and *-9* ‘D1 + GB’ and ‘D2 + GB’ plants as compared to ‘D1’ and ‘D2’ plants (Figure 5A).

To examine the relationship between water status and osmoprotectant levels, we measured Pro, TFAA, TSS, and Carb in barley leaves. (Figure 5B–E). In contrast to ‘CK’ plants, both barley varieties exposed to drought stress significantly enhanced Pro, TFAA, TSS, and Carb levels in the leaves (Figure 5B–E). In comparison to the respective ‘D1’ and ‘D2’ plants, ‘D1 + GB’ and ‘D2 + GB’ *BARI Barley-6* and *-9* plants demonstrated notable improvement in the levels of Carb and TSS, while decreasing the content of Pro and TFAA (Figure 5B–E). Moreover, only ‘GB-treated’ plants enhanced TSS and Carb content and showed a notable reduction in Pro and TFAA in both barley varieties in comparison to ‘CK’ plants (Figure 5B–E). The results of our research indicate that ‘GB-treated’ drought-tolerant *BARI Barley-9* plants had a substantial increase in leaf RWC, Pro, and TSS, and a decrease in the level of Carb content compared to drought-sensitive *BARI Barley-6* plants (Figure 5B–E).

## 4. Discussion

Drought is a major obstacle that threatens global agriculture by altering plant growth, development, and yield [37,38,39]. In particular, barley alone loses 44–48% of its grain yield under drought due to drought-induced disruptions to plant growth, photosynthesis, respiration, nutrient and hormonal balance, oxidative status, and osmotic regulation [40,41,42,43,44]. To maintain its productivity and minimize yield losses for food security, it is crucial to develop effective strategies to protect plants against drought stress. In modern agriculture, the application of exogenous molecules to improve plant stress responses plays a vital role, as these compounds are already compatible with plant systems. Considering the significant role of exogenous application of signaling molecules, we exogenously applied glycine betaine (GB) to two barley varieties to enhance their drought tolerance by investigating the underlying physiological and biochemical mechanisms.

Our findings indicate that water deficit conditions negatively altered the phenotypic appearances of both barley varieties (Figure 1A–D). The reduction can be attributed to several factors, including decreased water supply to the leaves from the soil, reduction in size of plant vascular tissues due to loss of turgor pressure, and dry matter accumulation [40,41,42]. Moreover, water-deprived conditions resulted in inhibition of ionic absorption and hormonal imbalances, which reduced turgidity, cell expansion, and photosynthesis, ultimately leading to leaf shrinkage and stunted growth and development in plants [43,44,45,46]. Conversely, supplementation of GB largely improved drought tolerance in barley varieties by inhibiting leaf wilting, suppressing drought-induced phytotoxic effects, as well as restoring greenness, and enhancing plants’ drought tolerance (Figure 1A–J). These endurances are likely to be associated with GB-mediated improvements in cellular water status, as evidenced by increased RWC along with increased growth in this study. Supporting this, the GB application promoted root length and biomass, an important indicator of improved developmental traits that enhance the plant’s ability to access water. Consistent with our findings, earlier research has also highlighted the effect of GB in promoting active growth recovery from drought in rice [47], safflower [48], oat [49], sweet potato [50], and watermelon [21].

Under water-deficit conditions, plants substantially fail to continue regular photosynthesis, which is the key mechanism for carbon fixation and biomass accumulation [51]. This reduction is due to both stomatal (lower CO_2_ diffusion into the leaf) and non-stomatal constraints (impairment of metabolic functions) [52]. When exposed to insufficient water, plants predominantly regulate ABA signals through xylem to the leaves, which stimulates stomata closure and decreases the CO_2_ influx and water transportation. Water scarcity leads to chloroplast membrane disruption, and the degradation of photosystem I and II (PS I and PS II), and inhibition of enzymes such as Rubisco and RuBP [42,52,53,54]. On the other hand, stomatal closure also helps limit water loss through transpiration and maintain leaf hydration, which is a drought-adaptation strategy. Therefore, plants face a trade-off between water loss and the maintenance of photosynthesis under water-deficit conditions. Our findings indicated that drought reduced photosynthesis, transpiration, and stomatal conductance, as well as levels of photosynthetic pigments, such as Chl *a*, Chl *b*, total Chls, and Caro, while increasing leaf temperature (Figure 2A–H). These perturbations hinder overall photosynthetic performance—resulting in growth arrest under two different drought conditions. Consistent with our findings, drought-induced attenuation of photosynthetic performance and degradation of photosynthetic pigments have also been recorded in other plant species [54,55].

In contrast, supplementation of GB to barley varieties under water deficit conditions effectively protected gas-exchange features and photosynthetic pigments in both varieties (Figure 2A–H). GB-mediated improvement in photosynthetic performance was associated with enhanced RWC. It is plausible that improved drought tolerance by GB supplementation is linked with increased water status in barley. Parallel to our findings, supplementation of GB has been shown to effectively increase chlorophyll content and maximize growth potential in diverse plant species, such as flax (*Linum usitatissimum*) [56] and kalmegh (*Andrographis paniculata*) [57] under drought. Our present findings, together with previous reports, suggest that GB helps maintain photosynthetic performance by balancing gas exchange, enhancing pigment content, and decreasing water loss to maintain RWC, even under insufficient soil water levels.

Under water deficit conditions, leaf functions are significantly impaired by disruption of electron flow during photosynthesis due to an imbalance between light capture and the utilization of plant photosystems. This imbalance leads to excessive ROS generation and oxidative damage in plants [58,59]. ROS directly attack membrane lipids and disrupt photosynthetic pigments, contributing to membrane disintegration, imbalanced cellular homeostasis, and even cell death. These effects ultimately stifle plant growth [41,42,60]. Our results demonstrated that barley leaves accumulated elevated O_2_^•−^, H_2_O_2_, and MDA, along with increased EL (Figure 3A–G), indicating that both barley varieties are subjected to severe drought-mediated oxidative stress and damage. Drought-induced oxidative stress has also been documented in various plant species, including wheat (*Triticum aestivum* L.) [61], cotton (*Gossypium hirsutum*) [59,62], pauciflora (*Calligonum mongolicum*) [60], and sesame (*Sesamum indicum* L.) [54]. GB-treated plants, on the other hand, diminished ROS accumulation by decreasing the levels of MDA, H_2_O_2_, and EL in barley leaves when subjected to drought stress (Figure 3E–G).

To counteract the severity of ROS-induced oxidative damage under drought stress, we examined the responses of the ROS-detoxifying antioxidant defense system. We measured the activities of APX, CAT, POD, and GST, along with the levels of total phenolic and flavonoid in the leaves of both barley varieties under drought conditions, with and without supplementation of GB (Figure 4A–F). Our results showed that plants under water-deficit conditions had increased activities of APX, GST, and POD, while CAT activity decreased (Figure 4A–D). GB-treated plants further enhanced these activities, likely contributing to the detoxification of the elevated ROS accumulation. APX, CAT, and POD are involved in detoxifying H_2_O_2_ [63], while GST (glutathione S-transferase) is a detoxification enzyme that neutralizes lipid peroxidation-derived toxic aldehydes, and this detoxification process leads to a reduction in lipid peroxides and reactive oxygen species (ROS) [64], in ‘GB-treated’ drought-stressed barley plants (Figure 4A–D). In Addition, plant secondary metabolites, including total phenolics and flavonoids, are recognized as non-enzymatic antioxidants that protect cell membrane integrity from ROS-induced oxidative damage during drought stress [65]. In this study, the high levels of total phenolics and flavonoids in GB-supplemented plants indicate that GB helps barley varieties protect against drought-induced oxidative damage (Figure 4E,F). The synergistic functions of both enzymatic and non-enzymatic defense systems against ROS likely contributed to improved growth in barley varieties treated with GB under water-deprived conditions.

Under drought stress, plants face osmotic pressure that causes a water imbalance in their cell [25,66]. To address this imbalance, plants produce various osmoprotectants, especially Pro, to maintain water balance and cellular integrity under stress [46,67]. Our results revealed that barley plants under water-deficit conditions showed increased levels of Pro compared to drought-free control plants (Figure 5B). Unlike the antioxidant defense, this increased Pro may not always be sufficient to maintain water status [38,68]. Conversely, GB supplementation decreased water loss and increased RWC in drought-stressed barley leaves without significantly increasing Pro levels (Figure 5A,B). This might be because GB effectively helps to restrain water loss from shoots without accumulating much Pro [69,70]. We also observed increased levels of TFFA, TSS, and Carb in drought-stressed barley leaves. Notably, GB-supplementation increased TSS, TFAA, and Carb levels under water-deficit conditions (Figure 5C–E). These results revealed that GB effectively reduces Pro and enhances the activity of osmoprotecants via cellular signaling, membrane protection, and metabolic biosynthesis and supply of organic nitrogen and carbon [56,63,71]. Previous studies have reported comparable findings on carpetgrass (*Axonopus compressus*) [68] and spinach (*Spinacia oleracea*) [70].

## 5. Conclusions

**Figure 6:**
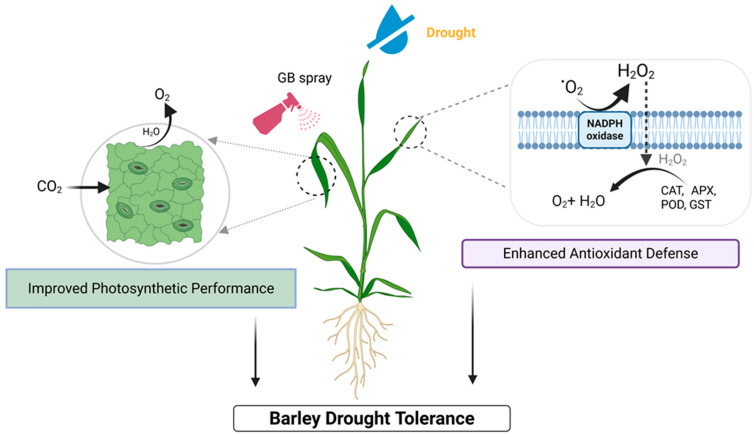
A model represents how exogenous Glycine betaine (GB), applied as a foliar spray, regulates different biochemical and physiological mechanisms to mitigate the effects of drought stress in barley. Under drought stress, foliar application of GB initiates dual protection pathways. Firstly, GB application leads to the improvement of photosynthetic performance by maintaining gas exchange and pigment levels in barley under water-deficit conditions. Secondly, GB enhances the antioxidant defense system to counteract the drought-induced accumulation of reactive oxygen species (ROS). Drought stress leads to excessive production of the superoxide radical (O_2_^•−^) and subsequent production of hydrogen peroxide (H_2_O_2_). GB mitigates oxidative damage by upregulating and enhancing the activities of key ROS-scavenging enzymes: catalase (CAT), ascorbate peroxidase (APX), peroxidase (POD), and glutathione S-transferase (GST). The synergistic effect of maintaining photosynthetic efficiency and detoxifying ROS ultimately leads to enhanced barley drought tolerance.

Our findings support that GB enhances defense responses in drought-stressed barley plants by influencing various physiological and biochemical processes (Figure 6). GB-sprayed plants exhibited increased root biomass, which helped maintain water and nutrient levels, delayed pigment degradation, and boosted photosynthetic activity and relative water content. Under drought conditions, GB effectively reduced drought-induced ROS by increasing enzymatic activities and non-enzymatic antioxidants, along with higher levels of soluble sugars, thereby contributing to osmotic adjustment. Thus, GB is crucial for improving barley’s drought tolerance. Future research should be conducted at the field-level trial, along with economic analyses, to validate GB’s potential to reduce drought-related crop losses.

## Data Availability

The datasets supporting the conclusions of this article are included within the article.
